# Evolutionary and molecular characteristics of high-Shannon entropy codons in VP1 of Coxsackievirus A6

**DOI:** 10.1099/jgv.0.002306

**Published:** 2026-07-29

**Authors:** Han Mo, Hui Li, Liu Yi, Ke Qian, Juan Lin, Ziqi Lin, Fenglan He, Xian Zhang, Xiansheng Ni, Xianfeng Zhou

**Affiliations:** 1 Evidence-Based Medical Research Center, Mass Spectrometry Research Center for Diagnosis, Treatment, and Chronic Disease Rehabilitation, Jiangxi University of Chinese Medicine, Nanchang, PR China; 2 Jiangxi Provincial Health Commission Key Laboratory of Pathogenic Diagnosis and Genomics of Emerging Infectious Diseases, Nanchang Center for Disease Control and Prevention, Nanchang, PR China; 3 Public Health Education Center, Jiangxi University of Chinese Medicine, Nanchang, PR China

**Keywords:** amino acid variation, capsid protein, co-evolution, CVA6, Shannon entropy, spatial conformation

## Abstract

Coxsackievirus A6 (CVA6) has emerged as a predominant global pathogen of hand, foot and mouth disease, yet its evolutionary and molecular characteristics remain poorly understood. This study integrated 10 year molecular surveillance data from central China (2013–2024) with global CVA6 sequences to analyse high-entropy codons in the VP1 capsid protein and their co-evolution patterns. Phylogenetic and Bayesian evolutionary analyses indicated that CVA6 strains in China primarily clustered into sub-genotype D3, with an estimated global VP1 substitution rate of 3.62×10⁻³ substitutions/site/year. Shannon entropy analysis identified eight high-Shannon entropy codons in VP1 (sites 5, 29, 30, 97, 137, 174, 242, 283), with site 283 exhibiting the highest variability. A novel 283V variant (*n*=12) was uniquely detected in China after 2018. Direct-coupling analysis suggested co-evolution between residue pairs, most notably VP1-137 and VP1-242 (Direct information=0.44), where S137N may form new hydrogen bonds with 243 h, potentially contributing to structural changes that could influence immune evasion. Furthermore, S97N formed hydrogen bonds with D99, thereby altering the BC loop region. A spatiotemporal analysis showed that 283A replaced 283T on a global scale after 2017, while minor variants (e.g. 30D, 97I, 174A) exhibited localized circulation. These findings highlight the predominance of high-entropy codons in VP1 surface loops (BC, DE, EF and HI), suggesting ongoing adaptive selection pressure. Continuous genomic surveillance is imperative for tracking emerging variants (e.g. 283V, 97N) and for informing vaccine design against evolving CVA6 strains.

## Graphical abstract

Molecular surveillance, phylogeny and co-evolution analyses suggest that high entropy codons in the CVA6 VP1 protein are associated with global genotype replacement and conformational changes, offering potential insights for vaccine design.

## Data availability

163 CVA6 VP1 sequences obtained in this study were deposited in GenBank (accession no. OL677506-OL677510, OL688664-OL688754, MW075633-MW075642, PP465653-PP465709).

## Introduction

 Hand, foot and mouth disease (HFMD) is a prevalent infectious disease that primarily affects young children aged 5 years or younger worldwide [1, 2]. HFMD is caused by human enteroviruses (EVs) and is characterized by the presence of fever and herpes-like vesicles on the hands, feet, mouth and buttocks [3]. Although the majority of cases are self-limiting and mild, some may rapidly progress to severe neurological and systemic complications, including aseptic meningitis, encephalitis and myocarditis and even death [4]. EVs are categorized into four species: EV-A, EV-B, EV-C and EV-D [5]. EV-A includes coxsackievirus group A (CVA) and a variety of EVs, which account for over 90% of HFMD cases. Among these, EV-A71 is a notable example. It was previously thought that EV-A71 and CVA16 were the predominant pathogens [6, 7]. Nevertheless, since the first reported Coxsackievirus A6 (CVA6) outbreak occurred in Finland in 2008, global molecular surveillance data have indicated a substantial shift in the pathogen spectrum [8]. Subsequent surveillance in many countries and regions has also confirmed a consistent high prevalence of CVA6, establishing it as a globally dominant pathogen of HFMD and herpangina [9–11].

CVA6 has been associated with a greater extent of skin lesions and more severe tissue destruction [2]. For instance, it can cause atypical clinical manifestations, such as eczema coxsackium, extensive erosions and vesicles, palmar and plantar desquamation and nail loss [12–14]. These atypical symptoms may persist longer than those of typical non-CVA6 HFMD [15].

CVA6 belongs to the species EV-A, genus *Enterovirus*, in the family *Picornaviridae* [16]. It is a single-stranded, positive-stranded, non-enveloped RNA virus with a genome of ∼7,400 nucleotides [8, 17]. The viral genome consists of a single open reading frame (ORF) that is flanked by 5′ and 3′ untranslated regions. The ORF is divided into three distinct regions: P1, P2 and P3. The P1 region encodes four structural proteins, designated as VP1-VP4. In contrast, the P2 and P3 regions encode seven non-structural proteins, denoted as 2A-2C and 3A-3D, respectively [18]. The *VP1* gene, which encodes major neutralizing epitopes, has been widely employed for genotyping and phylogenetic analysis [19].

In this study, we combined sentinel hospital-based EV molecular surveillance with global CVA6 strains to monitor and trace key mutations that may determine viral phylogeny. By aligning VP1 sequences of global CVA6 strains, we applied Shannon entropy analysis. Shannon entropy analysis that measures the degree of amino acid variability at each alignment position [20] has been widely used to identify evolutionarily variable sites in viral proteins, such as influenza A virus haemagglutinin [21], respiratory syncytial virus F protein [22] and the SARS-CoV-2 spike protein [23]. Using this method, it is determined that VP1-283 exhibited the highest entropy, followed by codons at positions 5, 29, 30, 97, 137, 174 and 242. Phylogenetic analysis suggests that the T283A substitution plays a pivotal role in shaping viral phylogeny. Subsequent analysis of the molecular structure of P1 indicated that T283A altered the spatial conformation by forming hydrogen bonds with VP3-57N and VP3-66R, which may potentially influence immunogenicity – a hypothesis that requires experimental validation. Furthermore, the high-entropy codons of VP1 and the entire P1 region were subjected to co-evolution analysis. The results suggested the presence of potential co-evolution position pairs in the P1 region, underscoring the importance of genomic surveillance for future vaccine development and prevention strategies. To the best of our knowledge, this is the first study to focus on the high-entropy codons of CVA6 VP1 and their associated co-evolution analysis, highlighting the need for ongoing genomic surveillance worldwide.

## Methods

### Sample collection and molecular identification

Clinical samples were obtained from the routine HFMD surveillance network from 2013–2024. A total of 6128 clinical specimens were collected from HFMD patients. Swabs were stored in dedicated Universal Transport Medium (Yocon, Beijing, China) for transport. Stool samples were diluted to a 10% suspension using MEMs. After thorough mixing, RNA was extracted from 200 µl of each clinical sample using the QIAamp Viral RNA Mini Kit (Qiagen, CA) according to the manufacturer’s instructions. The CVA10, EV-A71, CVA16 and CVA6 viruses were confirmed using commercial real-time PCR (RT‒PCR) kits (BioPerfectus, China) as previously described. Samples positive for universal EVs but negative for the above serotypes were named untyped EVs. The use and analysis of all samples in this study were approved by the ethics committee of the Nanchang Center for Disease Control and Prevention, and the procedures were performed according to the approved guidelines.

### Determination of the entire VP1 nucleotide sequence of CVA6

Viral RNA was extracted from CVA6-positive clinical samples using the QIAamp Viral RNA Mini Kit (Qiagen, CA). The entire VP1 gene of CVA6 was amplified using a PrimeScript One Step RT‒PCR Kit (Takara, Japan) with specific primers as previously reported [7]. The whole procedure has been described previously. PCR products were purified using the QIAquick PCR Purification Kit (Qiagen, Germany), and then amplicons were sequenced in both directions using the ABI 3730 Genetic Analyzer (Applied Biosystems, USA).

### Downloading of gene or genome sequences of global CVA6 strains

All kinds of length gene sequences of CVA6 strains were downloaded from the NCBI Virus database (https://www.ncbi.nlm.nih.gov/labs/virus/vssi/) according to the collection data as of 2024. A total of 12,158 sequences along with the metadata were downloaded. The VP1 sequences of CVA6 in GenBank were first filtered using the NCBI Virus online filtering system (https://www.ncbi.nlm.nih.gov/labs/virus/vssi/#/) to exclude vaccine strains and lab passaged strains. The complete or near full-length genomes (>7,300 bp) and VP1 gene sequences (915 bp, *n*=5,999) were used for evolutionary and molecular analysis in this study.

### Phylogenetic analysis

All complete VP1 sequences (915 bp, *n*=5,999) were used for amino acid entropy calculation. Representative CVA6 VP1 sequences (*n*=309) (Table S1, available in the online Supplementary Material) were selected from the database for phylogenetic analysis. Combined with the 163 VP1 full-length nucleotide sequences of CVA6 obtained in this study, a total of 472 sequences constituted the dataset used for genotyping and BEAST analysis. The sequences were aligned using the Clustal X programme. Phylogenetic trees were constructed by the neighbour-joining method after estimation of genetic distance using the Kimura two-parameter method. A bootstrapping test was performed 1,000 times.

### Bayesian evolutionary analysis

The global evolutionary dynamics of CVA6 were inferred based on the entire VP1 region. The Markov chain Monte Carlo (MCMC) method implemented in BEAST (v1.8.4) was used to estimate the divergence time, temporal phylogenies and rates of evolution [21]. Based on the strict molecular clock model and Bayesian skyline as the preferred population growth model, the posterior probability was analysed according to the Metropolis-Hastings-Green algorithm as the preferential population growth model, and a Bayesian MCMC run comprised 100 million generations to ensure that each parameter could converge. The output from BEAST was analysed using TRACER (v1.7.1) (http://beast.community/tracer) (with estimated sample size (ESS) values higher than 200). When the ESS is greater than 200, the iterative operation is considered to have converged. A maximum clade credibility (MCC) tree was constructed using TreeAnnotator, and the burn-in option was used to remove the first 10% of the sampled trees; the resulting tree was visualized using FigTree (v1.4.4).

To understand the evolution of CVA6 strains, we constructed the VP1 gene dataset of CVA6 virus from 1992–2024, including 163 Nanchang datasets obtained in this study (GenBank no. OL677506-OL677510, OL688664-OL688754, MW075633-MW075642, PP465653-PP465709) and 309 datasets downloaded from the NCBI database, including 136 sequences from China, 30 from Japan, three from South Korea, 11 from Thailand, 10 from India, 25 from Vietnam, 6 from Philippines, 48 from France, four from Spain, nine from the United Kingdom, three from the United States, 10 from Australia, 12 from Russia and two cases in Denmark (Table S1).

### Entropy of VP1 codons and physical property analyses of high-entropy codons

CVA6 amino acid sequences were aligned, and the amino acid substitution entropy values of each site of the sequences were analysed in the Shannon Entropy online analysis platform (https://www.hiv.lanl.gov/content/sequence/ENTROPY/entropy_one.html). The amino acid was considered to be highly variable when the substitution entropy value was greater than 0.6 [24]. To reduce the influence of highly similar sequences on site-wise entropy estimates, we additionally performed a sequence similarity-based reweighting analysis. For each sequence in the alignment, we counted the number of other sequences (including itself) with pairwise identity equal to or exceeding a given threshold (98% or 95%). The weight of a sequence was then defined as the reciprocal of that count. For each VP1 position, we calculated the weighted frequency of each amino acid by summing the weights of sequences carrying that amino acid and dividing by the total sum of all sequence weights. The Shannon entropy at the position was then recomputed using these weighted frequencies. This approach reduces the contribution of redundant, highly similar sequences while preserving information from more distinct sequences. SeqLogo plots of key amino acid sites were generated using WebLogo software (v2.031). The atomic model of the CVA6 VP0-VP1-VP3 complex (PDB ID: 5YHQ, 5XS4) was obtained from the Protein Data Bank (PDB, https://www.rcsb.org/) and was analysed in ChimeraX (v1.5) to analyse the physical properties of the high-entropy codons and perform model construction.

### Direct-coupling analysis of residue co-evolution

A computationally efficient implementation of direct-coupling analysis (DCA) was used to evaluate the accuracy of contact prediction. Mean-field DCA calculations were performed using the pydca Python package (v1.23), in accordance with the protocols detailed in the GitHub repository (https://github.com/KIT-MBS/pydca). The resulting correlations among amino acid compositions at different sequence positions can be exploited to infer spatial contacts within the tertiary protein structure [25]. True positive residue pairs were identified using pyDCA based on the criteria of being separated by at least four amino acids and having a distance less than 8 Å [26]. To avoid arbitrary parameter bias, pseudolikelihood maximization DCA (plmDCA) was used to validate the couplings obtained with mfDCA. Thus, we tested three independent settings: (1) Default mfDCA: our original method; (2) Default plmDCA as implemented in pydca; (3) low regularization plmDCA [27, 27], which is widely accepted in the DCA literature as the standard plmDCA setting.

## Results

### Molecular surveillance of CVA6 in Jiangxi during 2013–2024

**Fig. 1.**
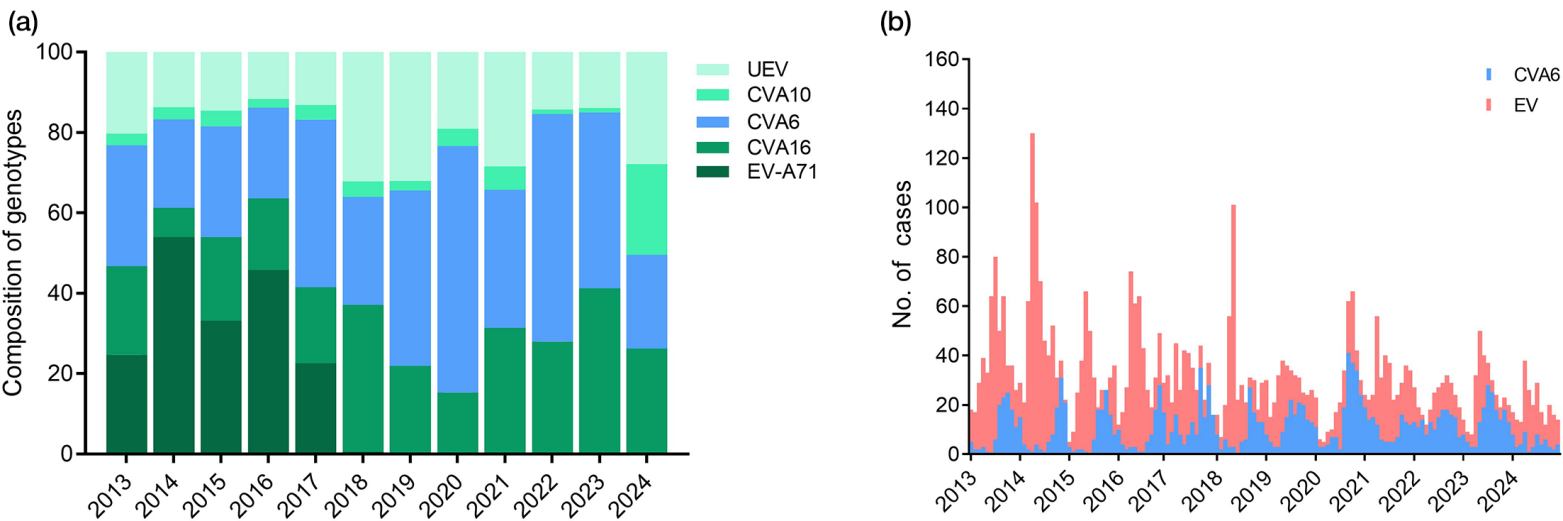
Molecular surveillance of EVs from HFMD cases in Nanchang, Jiangxi, China from 2013 to 2024. (a) Pathogen spectrum of EV-positive HFMD; (**b)** Monthly distribution of CVA6-positive and EV-positive cases.

Since CVA6 was added to the molecular surveillance of HFMD in 2013, it has persistently circulated in Nanchang, Jiangxi, China, accounting for 22.0–61.3% of EV-positive cases over the past decade (Fig. 1a). Following the disappearance of EV-A71 in HFMD cases in 2018, CVA6 and CVA16 began to co-circulate, underscoring the need for a tailored genomic surveillance strategy. Before 2020, the monthly distribution of CVA6-positive cases exhibited staggered peaks of CVA6 and total HFMD cases (Fig. 1b). In 2024, the number of CVA10 cases increased notably (Fig. 1a), highlighting the necessity for enhanced genomic surveillance for the emerging serotypes.

### Molecular epidemiology of CVA6 in Jiangxi, China

**Fig. 2.**
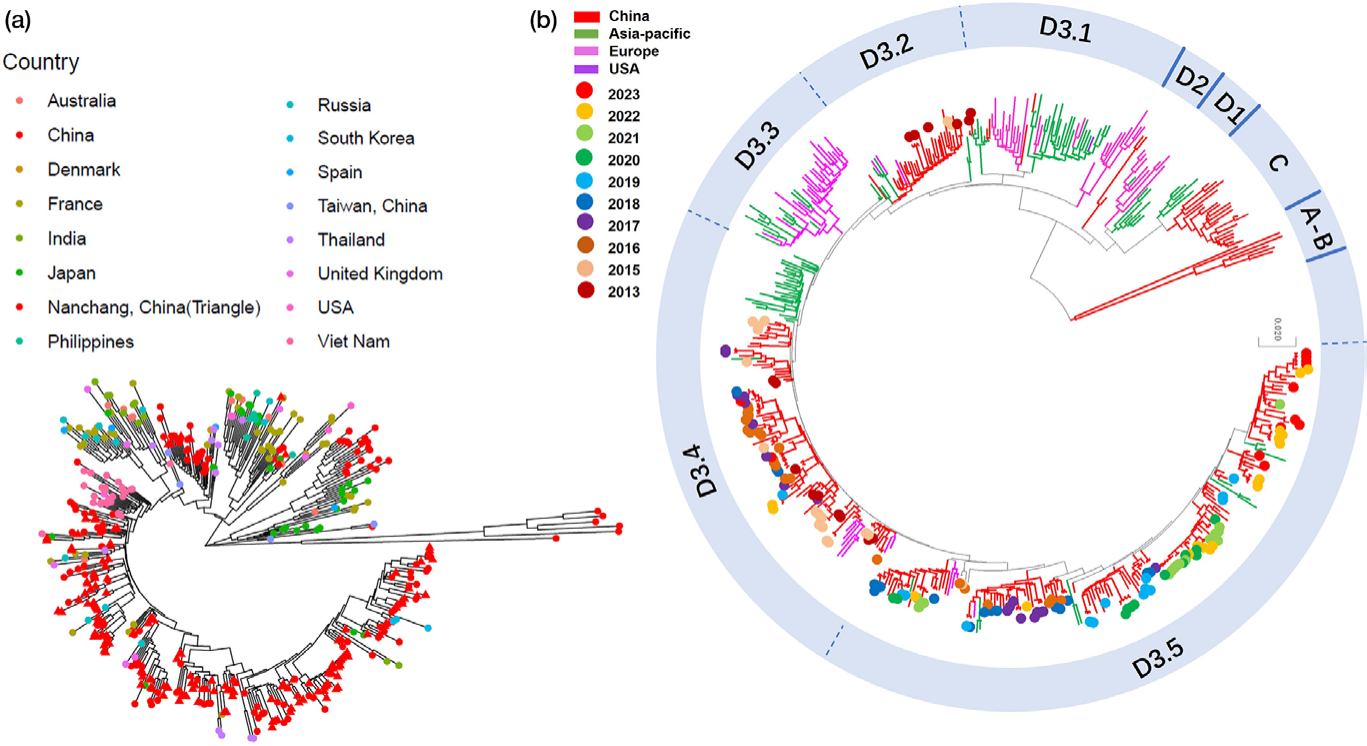
Neighbour-Joining tree of 163 local CVA6 strains and 309 representative strains around the globe. (a) Strains of origin were labelled with different colours; (**b)** Local strains collected during 2013– 2023 were labelled with dots.

**Fig. 3.**
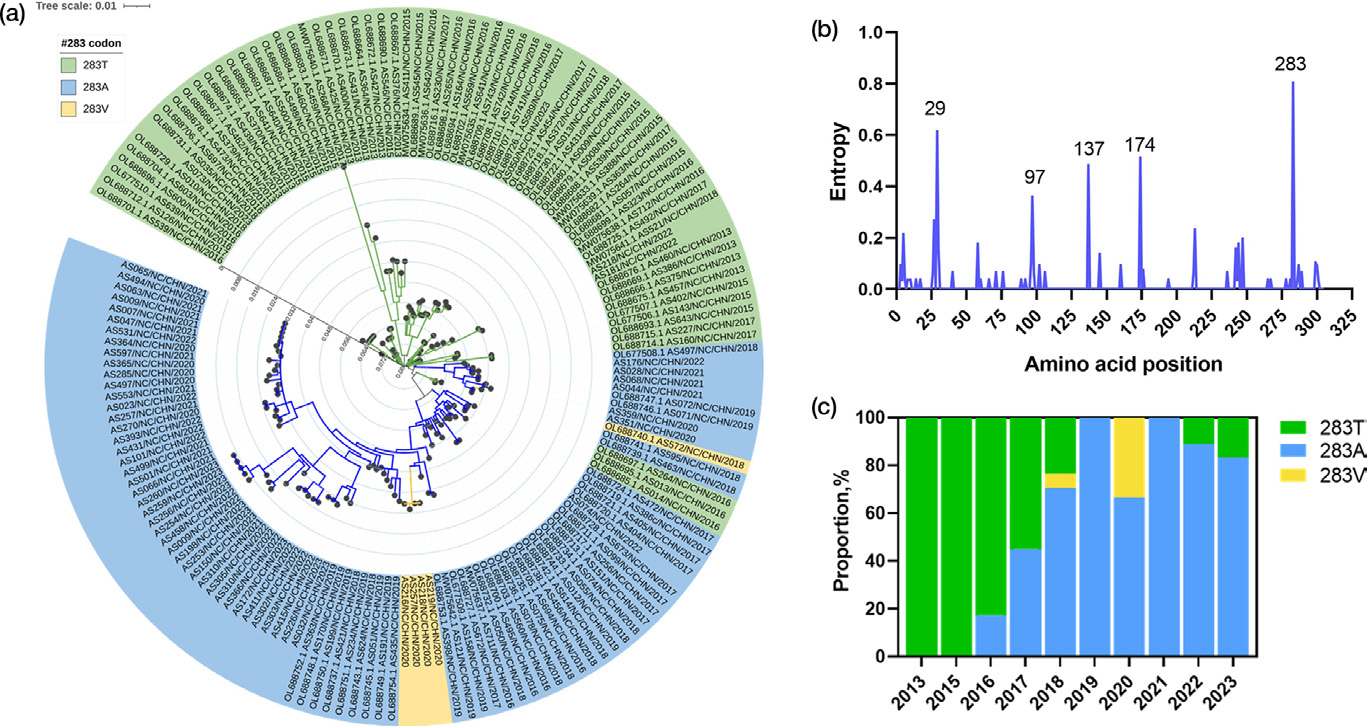
ML tree of CVA6 strains isolated in Nanchang, Jiangxi province, China from 2013–2023. (a) Full-length VP1-based phylogenetic tree of CVA6; (**b)** Entropy of 305 amino acid codons of VP1 protein; (**c)** Amino acids variability on the VP1-283 site of local CVA6 strains (*n*=163) in this study.

Phylogenetic analysis of the 163 representative strains collected from 2013–2023 revealed that they belonged to sub-genotype D3, which was further divided into four major subclades (D3.1, D3.2, D3.4 and D3.5) (Fig. 2). These strains showed nucleotide identities ranging from 87.5–100% and amino acid identities ranging from 96.3–100%. In subclade D3.1, two strains from Jiangxi were highly genetically similar to strains from the Asia-Pacific region. Phylogenetic analysis suggested that these strains likely originated from Europe. In subclade D3.2, several strains isolated between 2013 and 2015 were genetically similar to strains from mainland China, as well as to a few strains from Europe and the Asia-Pacific region. Most strains belonging to subclade D3.3 originated from Europe and the Asia-Pacific region; notably, no strains from China were found in this subclade. The majority of strains isolated from 2013–2016 were classified as subclade D3.4, which emerged from an outbreak in Vietnam in 2012. The strains circulating in Jiangxi evolved into several independent branches that co-circulated with mainland Chinese strains within subclades D3.4 and D3.5. This result indicated that locally circulating CVA6 strains have predominated in China since 2017. We also constructed a maximum likelihood (ML) tree of the CVA6 strains identified in this study and analysed the entropy of VP1 amino acids. The entropy value was highest at site 283, followed by sites 29, 97, 137 and 174 (Fig. 3b). Phylogenetic trees (Fig. 3a) were constructed to illustrate the amino acid variability at VP1-283. Analysis of global CVA6 strains (*n*=5,999) revealed a consistent spatial distribution trend of T283A.

### Bayesian evolutionary analysis

**Fig. 4.**
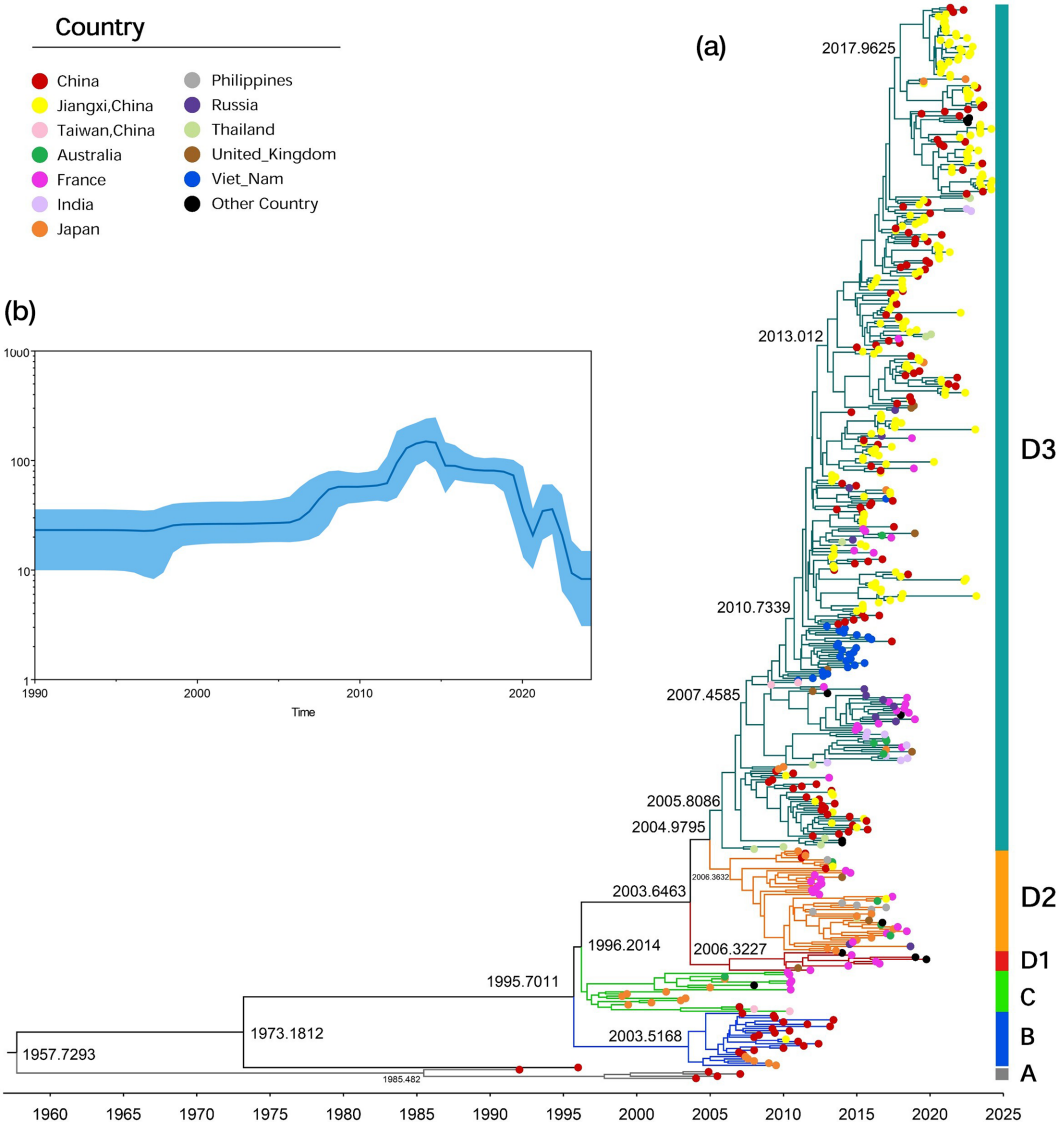
MCC tree and Bayesian skyline plot of the analysed CVA6. (a) MCC tree derived from the Bayesian analysis of the VP1 protein of CVA6 with the best fit model (strict molecular clock model), showing the tMRCA in each principal node. (**b)** Bayesian coalescent inference of genetic diversity and population dynamics using the Bayesian skyline plot available in BEAST 1.8.4. The X-axis represents the years of study, and the y-axis represents the relative genetic diversity product of the effective population size. The blue line represents the mean estimate, and the light blue shadow represents the 95% HPD.

To investigate the evolutionary history of CVA6, we constructed a MCC tree based on the complete VP1 sequences of representative CVA6 strains (Fig. 4a; Table S1). The average nucleotide substitution rate for the VP1 gene across global CVA6 strains was 3.62×10⁻³ substitutions per site per year (95% HPD: 3.34×10⁻³ – 3.95×10⁻³). Preliminary analysis indicates that the estimated time to the most recent common ancestor (tMRCA) of CVA6 is 1957.50, based on the absence of the prototype strain Gdula (AY421764). This date is close to the first documented detection of CVA6 in the United States in 1949.

The VP1 sequences of CVA6 have diverged into four distinct evolutionary branches, all of which originated from China. One branch emerged in 1985 as genotype A. Another notable branch**,** genotype B, emerged in 2003, with samples originating from China and Japan. The earliest C-genotype branch appeared in 1996, primarily from Japan but also from France, China, Taiwan, Australia and other regions. The estimated tMRCA of genotype D is 2003, and it is subdivided into three sublineages: D1, D2 and D3. Genotype D1 is predominantly found in France, the United Kingdom and other countries. Genotype D2 is present mainly in France and Japan, as well as in the Philippines, China, Australia, Russia, Thailand and elsewhere. Genotype D3 is the dominant branch in the phylogenetic tree, encompassing samples from all countries except the Philippines, with the highest number of samples originating from China. The strains from Jiangxi were predominantly concentrated in the D3 branch (Fig. 4a).

The Bayesian skyline plot, based on VP1 sequences of CVA6 sampled worldwide, revealed fluctuations in relative genetic diversity (RGD) from 2007 onward, peaking around 2014 (Fig. 4b), highlighting notable changes in viral diversity. This peak coincided with CVA6-associated HFMD outbreaks documented in Europe and the Asia-Pacific region. The RGD began to decline after 2015, with a sharp drop during 2018–2020. Following a rapid increase in 2021, it declined quickly and then levelled off in 2024.

### Evolutionary characteristics of high-entropy codons of global strains of CVA6

**Fig. 5.**
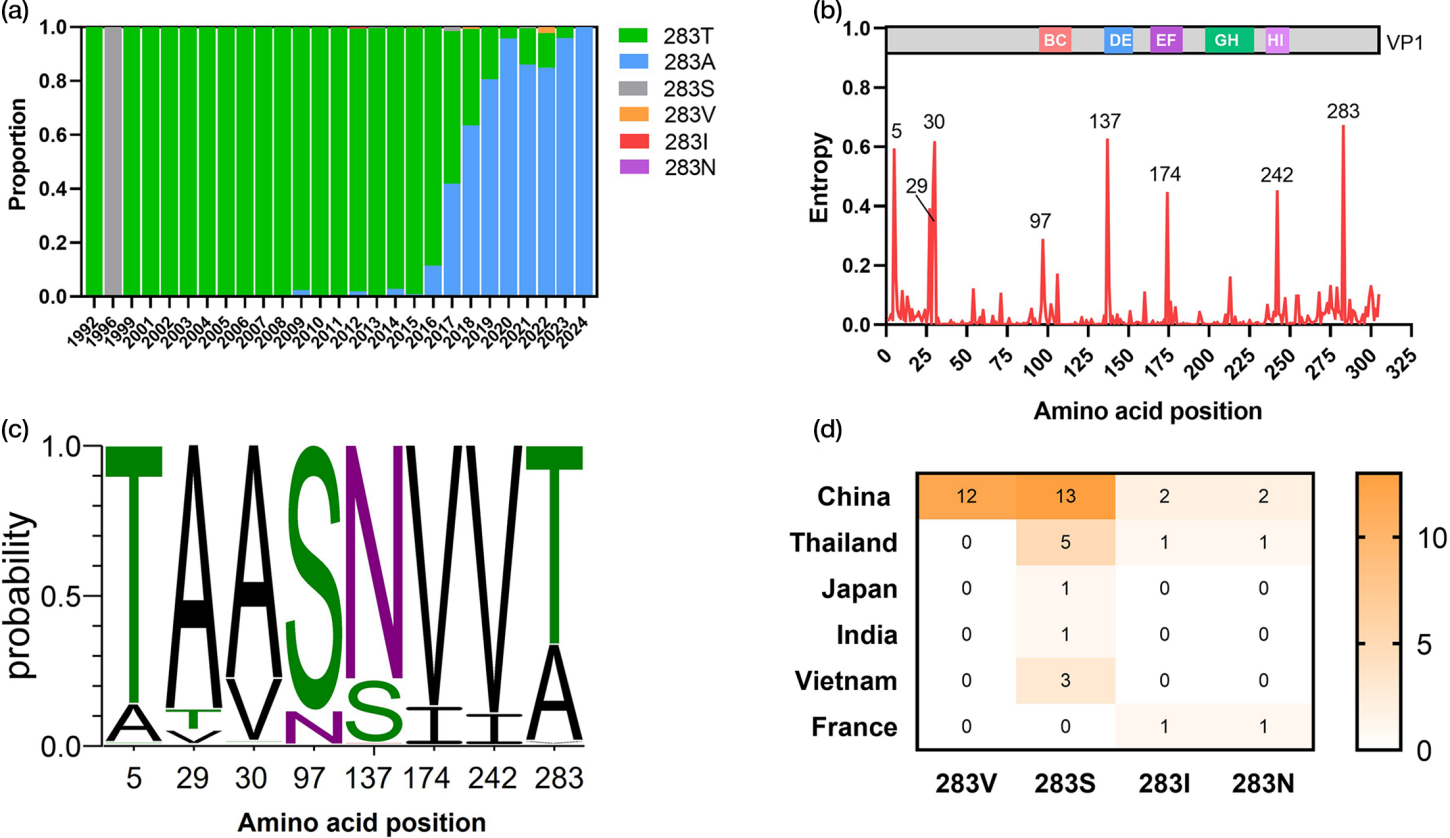
Amino acid variation of global CVA6 strains. (a) Entropy analysis of VP1; (**b)** Sequence logos for VP1 protein; (**c)** Yearly composition of amino acids on VP1-283; (**d)** The geographical distribution of the minor variants on VP1-283.

According to the results presented above, the complete VP1 (915 bp) or full-length genome sequences of global CVA6 strains were retrieved from GenBank. The analysis incorporated a total of 5,999 VP1 sequences derived from strains isolated from 1992–2024 (see Fig. 5a). Entropy analysis yielded a congruent outcome with that from our local strains (Fig. 5b), while WebLogo manifested the probability of each codon of high entropy (Fig. 5c). To assess whether the observed entropy was influenced by oversampling of phylogenetically related sequences, we performed a similarity-weighted entropy analysis. The effective sample size (*N_eff_*) decreased substantially under weighting (98% threshold: *N_eff_*=43.27; 95% threshold: *N_eff_*=8.39), indicating considerable sequence redundancy in the global dataset. Nevertheless, the eight high-entropy sites originally identified remained in the upper tail of the weighted entropy distribution (Fig. S2). For example, under 95% weighting, positions 29, 30, 97, 137 and 283 were still among the top 16 positions, suggesting that their high variability is not solely an artefact of over-sampling. Codon site 283 exhibited a distinct Thr to Ala substitution (T283A), exhibiting the highest entropy. However, sporadic strains were identified in a few countries, harbouring different amino acids in this codon (Fig. 5d). It was observed that CVA6 strains harbouring 283V (*n*=12) identified since 2018 exclusively circulated in mainland China, with five of these strains (41.7%) being isolated from Nanchang, Jiangxi province (Fig. 3a, c). The remaining cases were identified in Beijing (*n*=1, MZ516412), Guangzhou (*n*=1, MT119393) and Wuhan (*n*=5, OR507556-OR507558, OR507560-OR507561) in 2018, 2018 and 2022, respectively. This finding suggests that the substitution occurs via T283V or A283V. Specifically, the analysis indicates that strains harbouring 283T and 283A predominantly co-circulated when 283V emerged. It is imperative to closely monitor the 283V in subsequent surveillance operations.

**Fig. 6.**
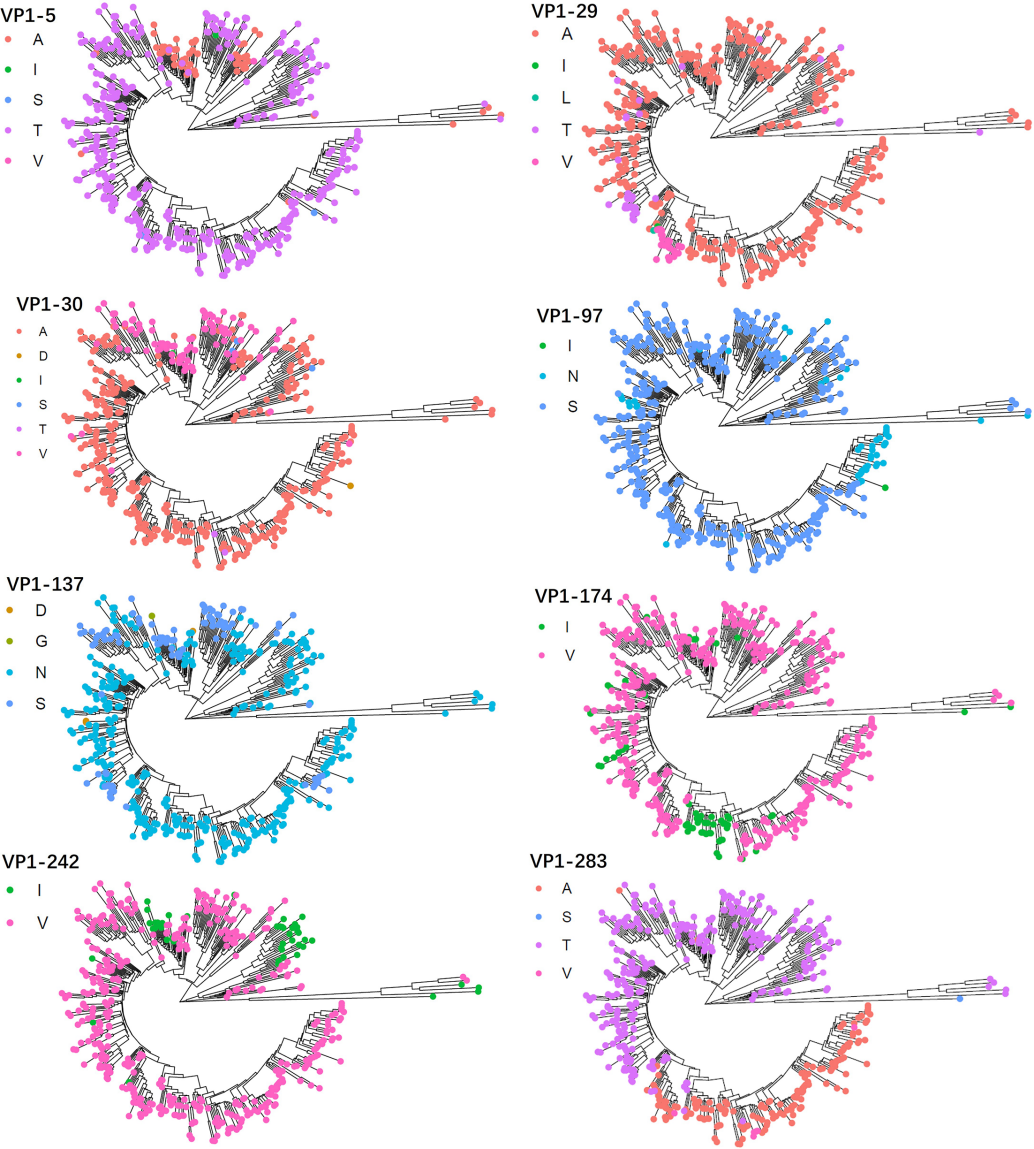
Phylogenetic trees of representative CVA6 strains around the globe. Amino acid variations of each high-entropy position (VP1-5, 29, 30, 97, 137, 174, 242, 283) were labelled with dots of different colours.

According to the phylogenetic trees with each high-entropy codon highlighted, it was found that isolates of genotype A and B harboured variations at sites 5, 29, 97, 174, 242 and 283 but not at sites 30 and 137 (Fig. 6). Overall, strains carrying the original amino acids at these sites predominated or co-circulated globally over the past 30 years (Fig. S1). However, several noteworthy findings emerged regarding high-entropy codons. First, one representative strain (GenBank No. PP191124.1) isolated from South Korea in 2022 exhibited a novel combination of 30D and 97I, suggesting the emergence of a distinct subclade in this region. Second, 97N has recently re-emerged in strains from China, including several local strains from this study. These Chinese strains showed high similarity to strains circulating in South Korea, raising the possibility that they may form a new subclade. Phylogenetic tree analysis indicated that 97N likely arose from 97S rather than being inherited from an earlier strain. Regarding site 174, strains carrying 174A (*n*=15) were identified exclusively in Hungary from July 2021 to March 2023, indicating restricted local circulation. Third, strains carrying 242V dominated globally, while 242I predominated in early CVA6 outbreaks in China. In the Philippines, a single strain carrying 242S was identified, with CVA6-242V remaining the predominant variant among submitted sequences (97.6%, *n*=85). Finally, 283T circulated predominantly worldwide before 2015. New variants carrying 283A emerged in 2009 and began to replace 283T on a global scale after 2017, as shown in Fig. 5a.

### Molecular characteristics of high-entropy amino acids in capsid protein

**Fig. 7.**
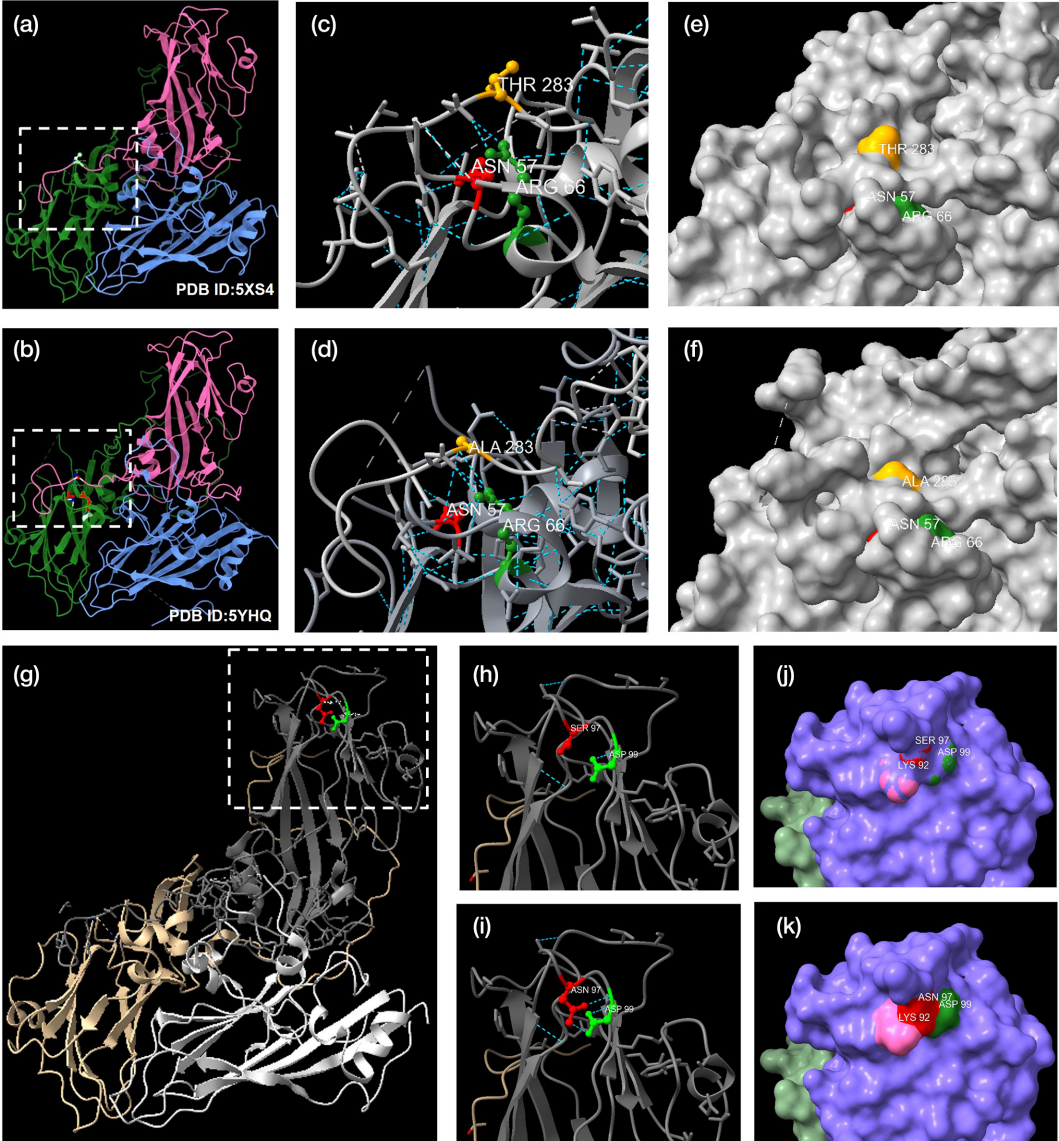
Spatial conformation with amino acid substitution at site 283 and 97. (a) VP0‐VP1‐VP3 complex (monomer, PDB ID: 5XS4); (**b)** VP0‐VP1‐VP3 complex (monomer, PDB ID: 5YHQ); (**c)** No hydrogen bond formation between VP1‐283T and VP3‐57N or VP3‐66R; (**d)** Hydrogen bond formation between VP1‐283A and VP3‐57N or VP3‐66R, respectively; (**e)** 3D structure with VP1-283T, VP3‐57N and VP3‐66R highlighted; (f) 3D structure with VP1-283A, VP3‐57N and VP3‐66R highlighted; (g) VP0‐VP1‐VP3 complex (monomer, PDB ID: 5XS4); (h) No hydrogen bond formation between 97S (red) and 99D (green); (i) Hydrogen bond formation between 97 (red) and 99D (green); (**j)** 3D structure with VP1-97S and 99D highlighted; (**k)** 3D structure with VP1-97N and 99D highlighted.

Structural analysis of the P1 molecule revealed that T283A alters the spatial conformation by forming hydrogen bonds with VP3-57N and VP3-66R, respectively (Fig. 7a–d). This hydrogen bonding partially reduces the exposure of residue 283, accompanied by increased bulging of surrounding amino acids (Fig. 7e, f). Furthermore, the conformational change may also result from the transition from hydrophilic (Thr) to hydrophobic (Ala). Taken together, T283A represents a candidate mutation that could be associated with CVA6 immunogenicity and pathogenicity, a hypothesis requiring future investigation. Additionally, S97N leads to the formation of two additional hydrogen bonds with VP1-99D (Fig. 7g–i). These bonds may induce a spatial conformation change near the BC loop, as shown in Fig. 7j, k.

As illustrated in Figs. 5, 12 CVA6 strains carrying 283V were isolated in China. A structural comparison of Val and Ala shows that they are similar in having an extra methyl group (CH_₃_). Specifically, Thr has an additional hydroxyl group (OH) and a methyl group, which contribute to its distinct chemical characteristics. The presence of the extra CH_₃_ group increases hydrophobicity at the protein level. This is exemplified by the observation that Val is more hydrophobic than Ala. Spatial resistance at the site may increase, and in the hydrophobic core, Val may provide stronger hydrophobic interactions, possibly as a result of adaptive selection.

### Co-evolution analysis

**Fig. 8.**
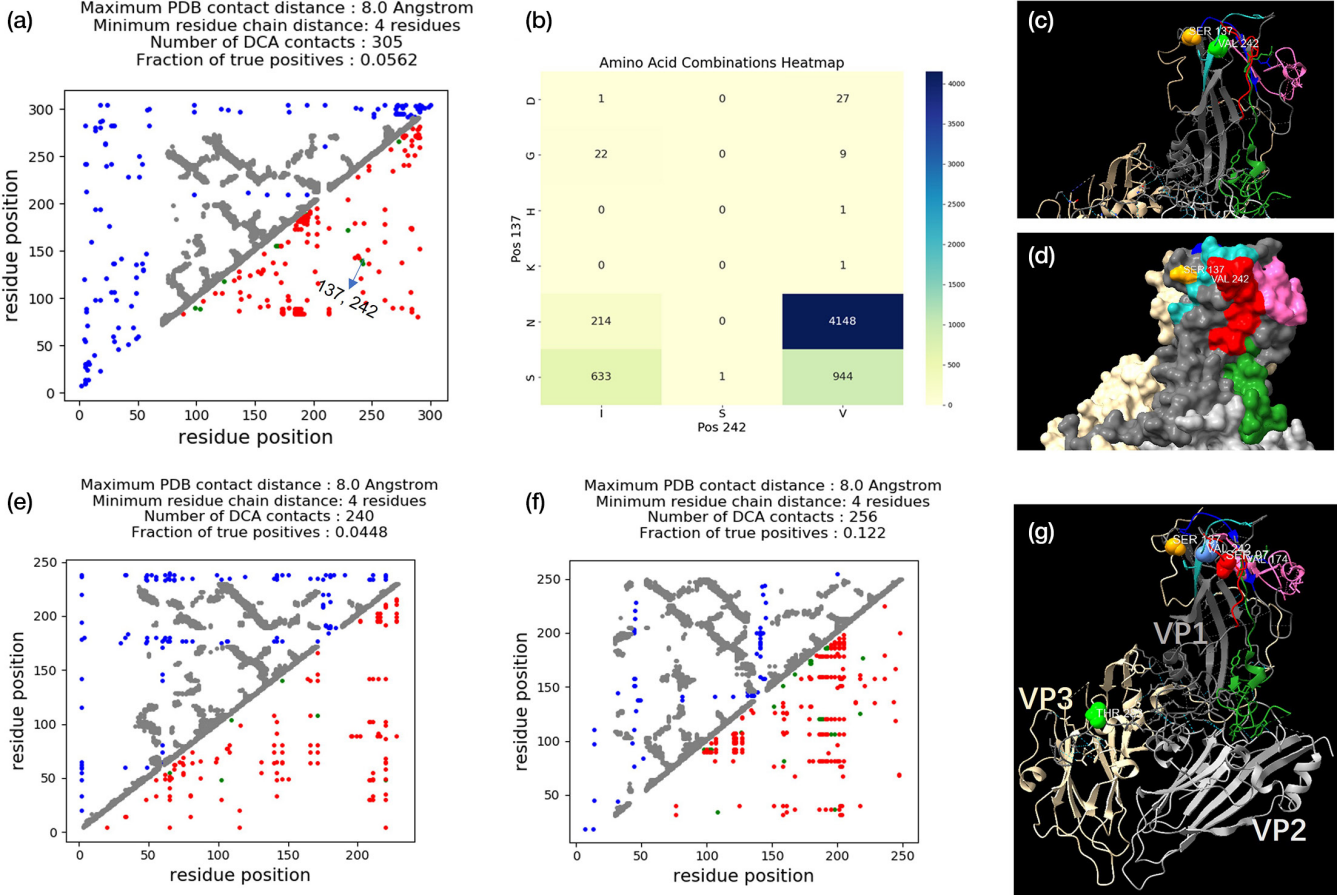
DCA of residue coevolution of capsid protein. (a) Comparison of pydca predicted contacts with PDB contacts in VP1; (**b)** Heatmap of DI value of position pairs of VP1;** (c)** Atomic position of 137 and 242 in spatial structure of VP1; (**d)** VP1-242 is unexposed to the surface of the protein; (**e)** Comparison of pydca predicted contacts with PDB contacts in VP3; (**f)** Comparison of pydca predicted contacts with PDB contacts in VP0; (**g)** High-entropy amino acids in spatial structure of P1. The grey dots are PDB contacts; others are predicted by pyDCA. The red dots are false positives, and the blue ones are due to missing residues in the PDB structure. The green dots are true positives. Red loop: BC, Dark blue loop: DE, Pink loop: EF, Green loop: GH, Light blue loop: HI.

**Fig. 9.**
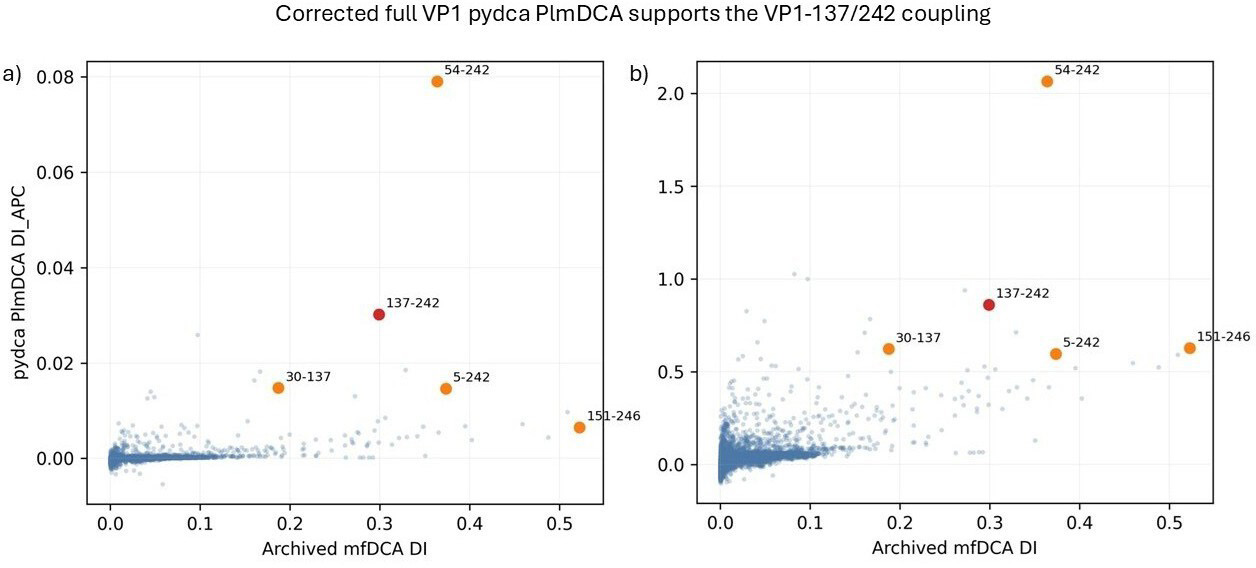
Full-VP1 plmDCA with corrected parameters supports the VP1-137/242 coupling. This pair ranks second in DI_APC (**a**) and fifth in FN_APC (**b**) among 45,451 pairs.

In this section, we present the visualization features of pydca by means of a visual comparison of the DCA-predicted contact maps with those obtained from the PDB structures for VP1 (PDB ID: 5YHQ, 5XS4). For this comparison, the top L DCA-ranked pairs were selected (Fig. 8a). The position pair 137–242 exhibited the highest direct information (DI) value of 0.44 and mutual information (MI) value of 2.12 in VP1 based on the mfDCA analysis (Fig. 8c), suggesting a potential statistical coupling between these two residues. Cross-validation using plmDCA with low regularization (λ=0.01) also ranked this pair among the top candidates (DI_APC rank: 2/45,451; FN_APC rank: 5/45,451) (Fig. 9), although the default plmDCA with strong regularization did not support it. Furthermore, sensitivity analyses incorporating sequence similarity weighting indicated that the ranking of pair 137–242 could be affected by over-sampled lineages (Fig. S3). The spatial configuration of CVA6 VP1 (Fig. 8d) shows that VP1-137 is exposed on the surface, while VP1-242 is buried within the structure. The DCA prediction was consistent with the heatmaps of amino acid variations at VP1-137 and VP1-242 (Fig. 8b) and the DI values of position pairs in VP1 (Table S2). Taken together, the 137–242 pair was supported by mfDCA and low-regularization plmDCA, but not by the default plmDCA in pydca. Hence, it is presented as a candidate coupling identified through multiple sensitivity analyses, not as a validated co-evolution event.

To further explore potential coupling pairs within the whole P1 region, DCA predictions for VP0 (Fig. 8e) and VP3 (Fig. 8f) were analysed, and a number of position pairs were observed (Table S2). Furthermore, most high-entropy codons were found to be concentrated within or in close proximity to the BC–HI loops, with the exception of VP1-283, which interacts with VP3 (Fig. 8g). Taken together, these results suggest the presence of candidate co-evolving residues in the P1 region, but the computational predictions remain sensitive to methodological parameters and sequence sampling. Therefore, experimental validation is required to confirm any functional role. Nonetheless, our findings highlight the value of continuous genomic surveillance for tracking emerging variants and informing vaccine development against evolving CVA6 strains.

### Spatial conformation with position pair 137-242 substitution

**Fig. 10.**
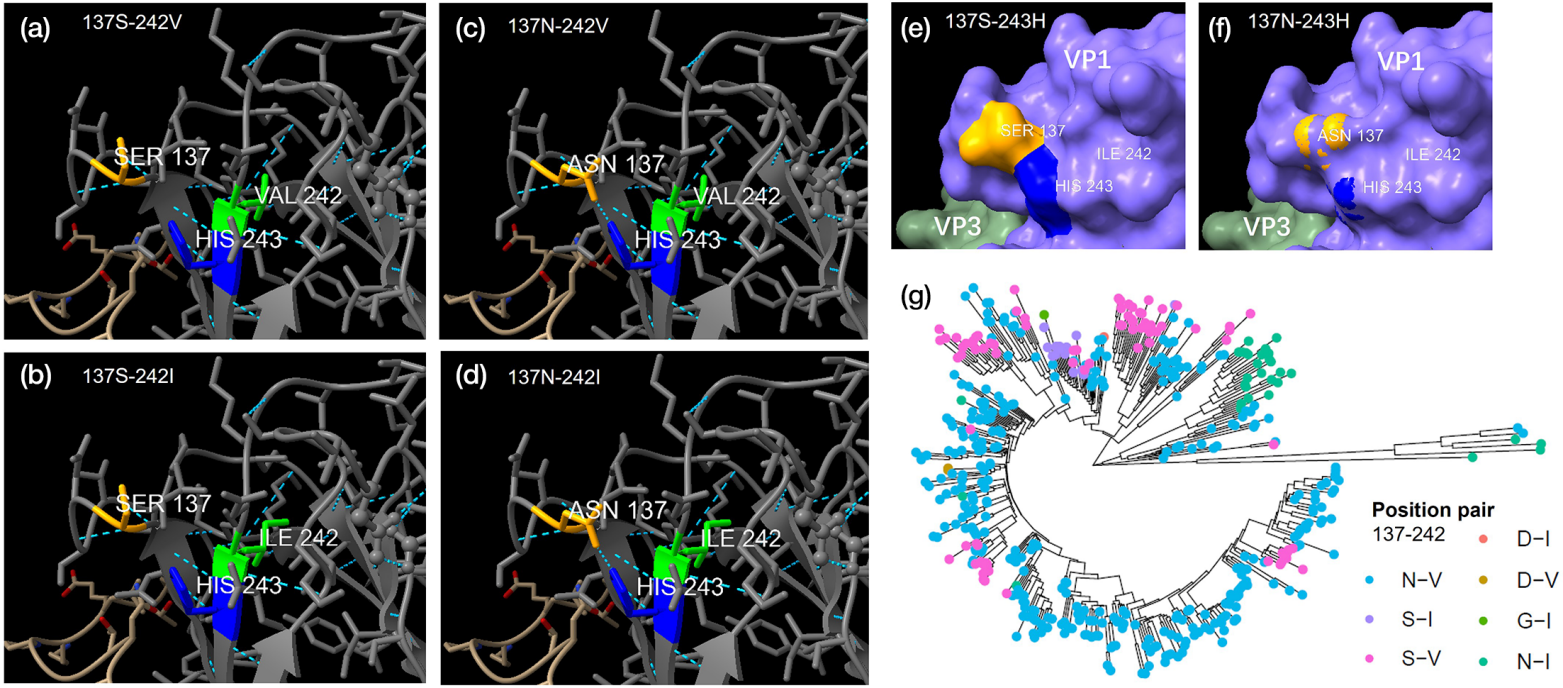
Spatial conformation with position pair 137–242 substitution. (a) Position pair 137S-242V;** (b)** Position pair 137S-242I; (c) Hydrogen bond formation between 137N and 243 h with position pair 137N-242V; (d) Hydrogen bond formation between 137N and 243 h with position pair 137N-242I; (e) No hydrogen bond between 137S and 243 h; (f) Hydrogen bond formation between 137N and 243 h; (g) Phylogenetic tree of representative CVA6 strains with position pair 137–242 labelled with different colours.

To further explore the molecular features of the potential statistical coupling between positions 137 and 242, we reconstructed the protein structure using the dominant substitution combinations at these two sites. The formation of a new hydrogen bond between 137N and 243 h was observed in the combinations 137S-242V (Fig. 10a), 137S-242I (Fig. 10b), 137N-242V (Fig. 10c) and 137N-242I (Fig. 10d). Consequently, an inward depression of these two amino acids is observed, accompanied by subtle alterations in spatial structure (Fig. 10e, f). These observations suggest that the S137N-associated spatial change may potentially influence the immunogenicity of the virus and could contribute to potential structural changes that may affect antibody recognition, but this remains a hypothesis that requires experimental validation. According to the phylogenetic tree of the worldwide representative strains, strains harbouring 137N-242V were predominant in D3.3–3.5 subclades, while strains with 137S-242V, 137N-242I, 137S-242I and 137N-242V co-circulated globally in early years (Fig. 10g).

## Discussion

CVA6 has become a predominant agent globally, particularly in Asia-Pacific [28–31] and Southeast Asia [32–35], and Europe [11, 36]. CVA6 mainly affects children under 5 years of age, and is more likely to infect adults than other serotypes [37]. Research on CVA6 vaccine development is limited, even though different vaccine platforms have been tried, including mRNA vaccines [18]. Traditional inactivated vaccines have limitations due to viral evolution and genetic recombination. Molecular characterization of the major capsid protein is vital to overcome the barriers to developing an ideal vaccine. Although the evolutionary dynamics of CVA6 have been previously clarified, the dynamic molecular characteristics of the CVA6 capsid protein are still unclear [9]. In this study, we for the first time characterized the co-evolution of position pairs in the capsid protein of CVA6, focusing particularly on the high-entropy codons of VP1. We conducted a comprehensive molecular surveillance study spanning a decade, encompassing both regional and global CVA6 strains. This extensive investigation identified numerous high-entropy codons, predominantly located within the BC, DE, EF and HI loops of VP1. A notable finding was a predominant T283A substitution, which emerged as the dominant variant between 2016 and 2019. The T283A-derived spatial conformation change could potentially affect the immunogenicity and pathogenicity of CVA6; however, direct experimental evidence is currently lacking. It is noteworthy that 12 strains harbouring 283V were exclusively identified in China, thereby emphasizing the necessity for enhanced molecular surveillance.

Proteins are composed of smaller molecules, known as amino acids, which are linked sequentially to form a polymeric structure. The arrangement of amino acids in a protein dictates its shape and, consequently, its function [38]. To the best of our knowledge, this is the first study to explore the potential co-evolution of CVA6 capsid protein. A number of candidate co-evolving position pairs were identified by DCA (Table S2), among which the VP1-137/242 pair (both high-entropy sites) was systematically analysed as a prioritized statistical coupling. The results indicated that S137N caused a spatial conformation change that might cause potential structural changes that may affect antibody recognition. Furthermore, S97N led to the formation of two additional hydrogen bonds with VP1-99D, resulting in a spatial conformation change near the BC loop. When considered collectively, the formation of hydrogen bonds resulting from amino acid substitutions has the potential to induce immunogenicity variations. The mean nucleotide substitution rate for the VP1 gene in CVA6 strains on a global scale was ∼3.62×10^–3^ substitutions/site/year. Constant molecular surveillance will facilitate the study of evolutionary dynamics and the development of vaccines. Furthermore, the evolutionary dynamics of high-entropy codons necessitate meticulous observation to address the following concerns: It is imperative to ascertain whether CVA6 strains harbouring 283A will predominate in the future or if CVA6 strains harbouring 283V will circulate beyond the confines of China.

Notwithstanding the pioneering nature of this study as the first to investigate amino acid co-evolution in CVA6 capsid proteins, several limitations must be acknowledged. First, one limitation of our entropy analysis is that sequences were treated as independent observations, whereas viral sequences are phylogenetically related. Although our similarity-weighted sensitivity analysis confirmed that the main high-entropy sites remain variable after correction, absolute entropy values may be influenced by uneven sampling. Future studies employing phylogenetic-based entropy corrections could further refine these estimates. Second, spatial conformation based on PDB data has its limitations when it comes to analysing the impact of multi-codon mutations on structural change. Finally, the potential effects of the identified mutations on immunogenicity or pathogenicity remain ambiguous in the absence of *in vitro* and *in vivo* experiments. Consequently, any functional interpretation presented in this study should be considered hypothesis-generating, and future investigations employing reverse genetics or neutralization assays are necessary to validate these hypotheses.

In summary, the present study conducted an in-depth analysis of the evolutionary and molecular characteristics of CVA6 capsid protein, offering a foundation for future studies of viral evolution, immunogenicity and pathogenicity in EVs. DCA has recently been applied to other RNA viruses, such as SARS-CoV-2, where it revealed epistatic interactions among spike mutations that change over time as mutations fix or disappear from the global population [39]. Moreover, in adeno-associated virus, a DCA-derived metric was found to correlate with viral transduction ability, suggesting that coevolutionary models can elucidate complex capsid residue interaction networks essential for viral function [40]. As noted above, high-entropy codons were predominantly located within or near predicted epitope loop regions, which suggests a hypothesis that these sites may be under selective pressure; however, experimental validation is required to determine any actual role in immune evasion. These findings contribute to our understanding of the evolutionary dynamics, co-evolution and spatial conformational change of the antigen, while all functional claims regarding immunogenicity or immune escape remain hypothetical until experimentally validated.

## Supplementary material

10.1099/jgv.0.002306 Supplementary Material 1.

10.1099/jgv.0.002306 Supplementary Material 2.

## Abbreviations

CVA6: coxsackievirus A6
DCA: direct-coupling analysis
DI: direct information
ESS: estimated sample size
EV: enterovirus
HFMD: hand, foot and mouth disease
MCC: maximum clade credibility
MCMC: Markov chain Monte Carlo
MI: mutual information
ML: maximum likelihood
ORF: open reading frame
PDB: Protein Data Bank
plmDCA: pseudolikelihood maximization DCA
RGD: relative genetic diversity
RT-PCR: real-time PCR
tMRCA: time to the most recent common ancestor
