# Supplementary material for: Evolutionary and molecular characteristics of high-Shannon entropy codons in VP1 of Coxsackievirus A6
Source: J Gen Virol. 2026 Jul 29;107(7):002306. doi: 10.1099/jgv.0.002306 (PMC13418908; doi:10.1099/jgv.0.002306)

Supplementary Figure S1

Yearly composition of amino acid variants on high-entropy codons.

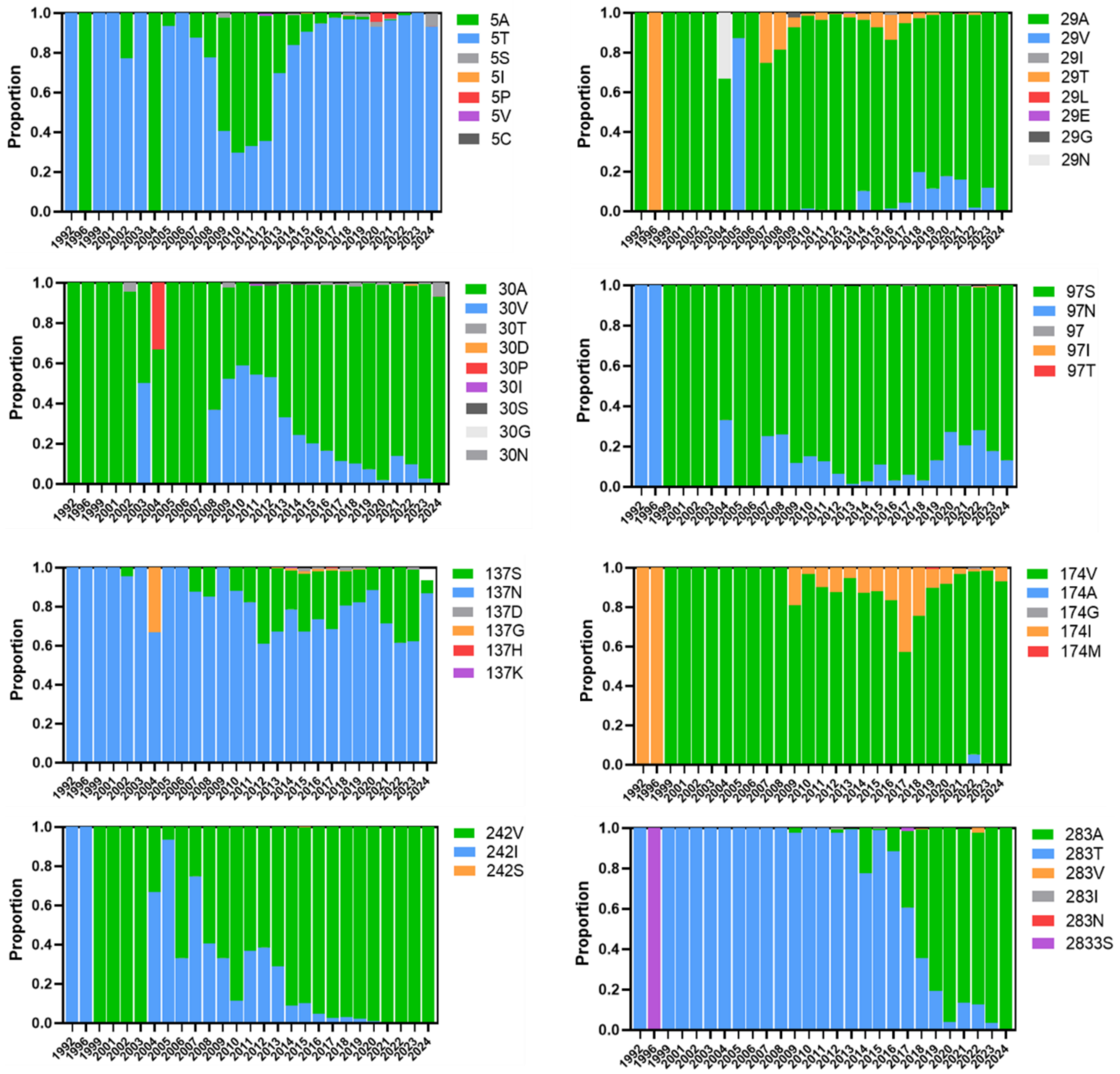

Supplementary Figure S2

Sensitivity analysis of VP1 entropy to sequence similarity weighting. The analysis compares the entropy of top-ranked VP1 sites under three conditions: unweighted, 98% weighted, and 95% weighted.

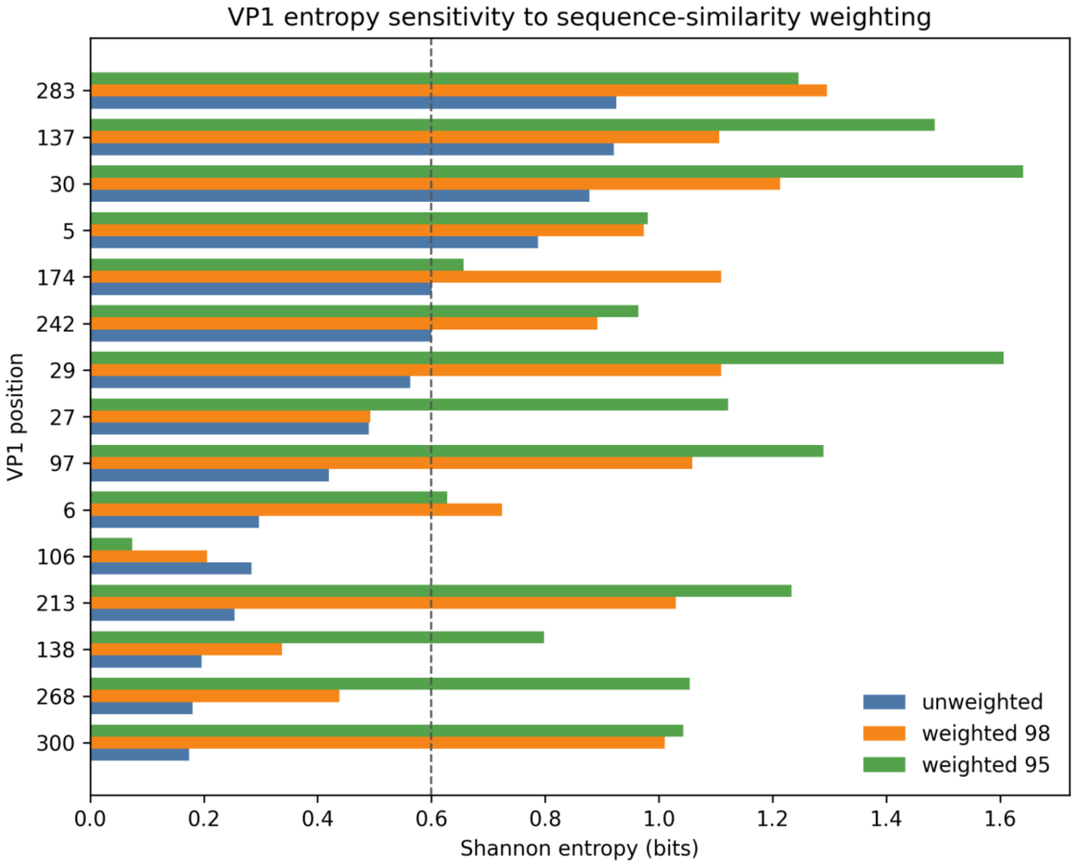

Supplementary Figure S3

Selected-site PLM-style weighted sensitivity analysis. The ranking of VP1-137/242 decreased after applying 98% and 95% sequence similarity weighting.

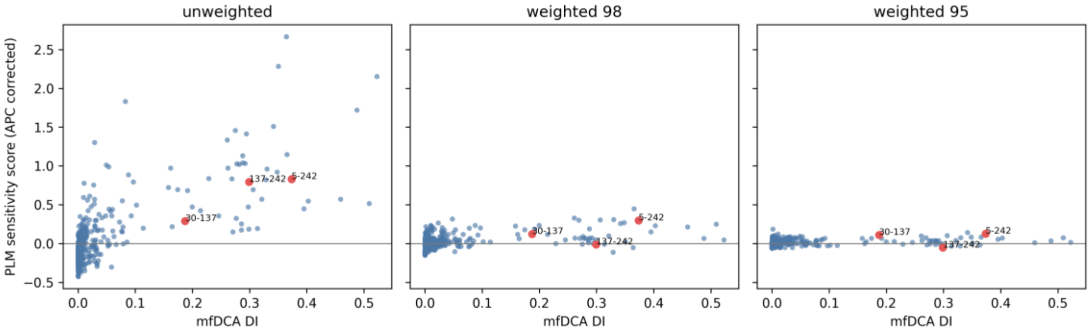

Supplement: Supplementary Material 1. [file jgv-107-02306-s001.pdf]
